# Diagnosis and Surgical Treatment of Hepatic Hydatid Disease

**DOI:** 10.1155/1992/41956

**Published:** 1992

**Authors:** Ahmet Bilge, Erdoğan M. Sözüer

**Affiliations:** Department of Surgery Erciyes University Medical School Kayseri Turkey

## Abstract

In this report two hundred and twenty six patients with hydatid disease were admitted to the Surgical Department of Erciyes University (Kayseri) and Şişli Etfal Hospital (Istanbul) between 1978 and 1990 and reviewed retrospectively. One hundred and two patients (45.1%) were male and 124 (54.9%) female. In the patients with hydatid cysts the most frequent symptom was right upper abdominal pain (66%). The most frequent signs were hepatomegaly (43.8%) and palpable mass (39%). One hundred and sixty seven patients (73.9%) were examined with ultrasonography which has a diagnostic value of 94%. Preoperative complications were infection of cyst (7%), intrabiliary rupture (3.5%) and anaphylactic shock (0.4%). All patients were operated on by using various surgical techniques; omentoplasty (101), external drainage of residual cavity (64), marsupialization (25), capitonnage (15), introflexion (10), pericystectomy (6), and hepatic resection (5).

The main postoperative complications were wound infection (12%) and biliary fistula (2.6%). The total mortality rate was 1.8% in this series.


*HPB Surgery*, 1992, Vol. 6, pp. 57–64
Reprints available directly from the publisher
Photocopying permitted by license only

© 1992 Harwood Academic Publishers GmbH
Printed in the United Kingdom

### DIAGNOSIS AND SURGICAL TREATMENT OF HEPATIC HYDATID DISEASE

AHMET BILGE and ERDOĞAN M. SÖZÜER
*Department of Surgery, Erciyes University Medical School, Kayseri, Turkey*

*(Received 30 July 1990)*

In this report two hundred and twenty six patients with hydatid disease were admitted to the Surgical Department of Erciyes University (Kayseri) and Şişli Etfal Hospital (Istanbul) between 1978 and 1990 and reviewed retrospectively. One hundred and two patients (45.1%) were male and 124 (54.9%) female. In the patients with hydatid cysts the most frequent symptom was right upper abdominal pain (66%). The most frequent signs were hepatomegaly (43.8%) and palpable mass (39%). One hundred and sixty seven patients (73.9%) were examined with ultrasonography which has a diagnostic value of 94%. Preoperative complications were infection of cyst (7%), intrabiliary rupture (3.5%) and anaphylactic shock (0.4%). All patients were operated on by using various surgical techniques; omentoplasty (101), external drainage of residual cavity (64), marsupialization (25), capitonnage (15), introflexion (10), pericystectomy (6), and hepatic resection (5).

The main postoperative complications were wound infection (12%) and biliary fistula (2.6%). The total mortality rate was 1.8% in this series.

KEY WORDS: Hepatic hydatid cyst disease, omentoplasty, introflexion

### INTRODUCTION

Hydatid disease of the liver remains an important worldwide health problem, including Turkey. It is especially endemic in many sheep grazing areas. As a result of the ease of travel and migration, the disease is now being encountered in immigrant adults in many parts of the world in which it is not endemic[1,2]. Hydatid cysts in the human liver occur when man becomes an accidental intermediate host for the larval form of *Echinococcus granulosus*, which lives as an adult worm in the canine intestine. The liver is the most common site infected in the adult. About one-third of patients with liver hydatid cysts have other sites involved, including peritoneum, lungs, spleen, brain, bone and thyroid.

In this study, the various surgical techniques were compared with regard to postoperative complications, hospital stay, return to activity and convalescence time. Herein, we discuss our experience over a 12 year period.

### PATIENTS AND FINDINGS

During the years 1978–90, 226 patients with hepatic hydatid disease underwent surgical treatment at the Surgical Department of Erciyes University and Sisli Etfal

Address correspondence to: Ahmet Bilge, Department of Surgery, Medical School, Erciyes University, Kayseri, Turkey.

Hospital (Istanbul). Patients' ages ranged from 15 to 75 (median 49) years. One hundred and two patients (45.1%) were male and 124 (54.9%) female.

### *Symptoms and Signs*

Symptoms arise from local pressure, leakage, infection and rupture into the biliary tree. Right upper abdominal pain, hepatic enlargement, and palpable mass were the most common signs of the disease. Pain may result from stretching of the liver capsule or leakage of contents. Leakage may also produce urticeria, or anaphylaxis when cyst fluid is absorbed into the blood stream. The duration of symptoms and signs ranged from 2 weeks to 8 years. The most common clinical findings are shown in Table 1.

### *Diagnosis of the Disease*

The diagnosis of hydatid disease of the liver is usually easily made when a liver cyst is found in a patient from an endemic area. Calcification of the cyst wall is commonly seen on plain X-ray films. Ultrasound showed the characteristic picture of a cyst containing multiple daughter cysts.

Out of twenty-eight patients who had radionuclide scanning of the liver, lesions were confirmed in all of them. Ultrasonographic examination was used in 167 patients and demonstrated hepatic cysts with 94% accuracy (Table 2). Ultrasound and CT scanning were also accurate in locating the site, size and number of intrahepatic cysts, together with the contained daughter cysts. Serological studies using the Casoni's skin test and Weinberg's test yielded poor results in patients tested.

**Table 1** Clinical findings in 226 patients with hydatid cysts of the liver

| *Clinical findings* | *n(%)* |
|---|---|
| Right upper abdominal pain | 149(66) |
| Hepatic enlargement | 99(43.8) |
| Palpable mass | 88(39) |
| Anorexia, weight loss | 57(25) |
| Nausea and vomiting | 48(21) |
| Fever and chills | 27(12) |
| Dispnea | 23(10) |
| Jaundice | 7(3.1) |
| Persistent cough | 5(2.2) |
| Urticeria and anaphylaxis | 1(0.4) |

**Table 2** Diagnostic procedures and results

| *Diagnostic procedure* | *n* | *Positive result(%)* |
|---|---|---|
| Ultrasound examination | 167 | 157(94) |
| Plain X-ray | 226 | 84(37.1) |
| Casoni's skin test | 62 | 42(67.7) |
| Weinberg test | 56 | 31(55.4) |
| Radionuclide scan | 36 | 36(100) |

Preoperative complications found in 25 patients were infection of cyst, intrabiliary rupture and anaphylactic shock (Table 3).

### *Surgical Techniques*

Cysts were mainly found in the right lobe (76%). Only 24% of cysts were localised in the left lobe. Of hundred and forty seven (65%) of patients who had a single cyst, 38 (16.8%) had 2, 27 (12%) had 3 cysts and 14 (6.2%) of patients had 4 or more cysts. In order to compare the results, in this study the patients are best analysed in two groups: uncomplicated cysts 201 patients (89%) and complicated cysts 25 patients (11%).

**Table 3** Preoperative complications of the liver cysts

| *Complication* | *n(%)* |
|---|---|
| Infection of cyst | 16(7) |
| Rupture into biliary tract | 8(3.5) |
| Intraperitoneal rupture | 1(0.4) |
| Total | 25(11) |

### *Uncomplicated Cysts*

In this group, the residual cavity was filled with pedicled omentum in 87 and with liver tissue in 25 patients. The cavity was simply drained in 79 patients (Table 4). In this group 33 patients developed the various complications shown in Table 5.

**Table 4** Management of the patients with uncomplicated cysts (n = 201)

| *Treatment* | *n* |
|---|---|
| Omentoplasty | 87 |
| External drainage of residual cavity | 64 |
| Marsupialization | 15 |
| Capitonnage | 15 |
| Introflexion | 10 |
| Pericystectomy | 6 |
| Hepatic resection | 4 |

**Table 5** Postoperative complications in patients with uncomplicated cysts ($n$ = 201)

| *Complication* | *n(%)* |
|---|---|
| Wound infection | 24(12) |
| Infection of residual cavity | 3(1.5) |
| Biliary fistula | 3(1.5) |
| Pulmonary infection | 3(1.5) |
| Total | 33(16.4) |

*Complicated Cysts*

In this group biliary drainage was added to omentoplasty in 8 patients with intrabiliary rupture. In 16 patients with infected cyst was drained like liver abscess (Table 6). The rate of complication was very high.

Postoperative morbidities such as: mean postoperative hospital stay and return to daily activity, period of drainage were found significantly longer in the complicated group than the uncomplicated group (Tables 8 and 9).

Four patients have died due to myocardial infarction (2) and renal failure (2). The mortality rate was 1.8% in this series. The follow-up period ranged from 3 months to 5 years (average 2.8 years). No patient underwent late surgery for reasons related to hydatid disease.

**Table 6** Surgical techniques in complicated cysts (n = 25)

| | *Complication and surgical procedures* | *n(%)* |
|---|---|---|
| Intrabiliary rupture (8 patients) | | |
| | Omentoplasty + T tube | 5 |
| | Omentoplasty + Colecystectomy + Cholodocoduodenostomy | 2 |
| | Omentoplasty + Colecystectomy + Sphincteroplasty | 1 |
| Infection of cysts (16 patients) | | |
| | Marsupialization | 10 |
| | Omentoplasty | 6 |
| Intraperitoneal rupture (1 patient) | | |
| | Hepatic resection | 1 |

**Figure 7** Postoperative complications in 25 patients with complicated cysts

| *Complication* | *n(%)* |
|---|---|
| Wound infection | 3(12) |
| Biliary fistula | 3(12) |
| Infection of residual cavity | 2(8) |
| Pulmonary infection | 2(8) |
| Intraabdominal abscess | 1(4) |
| Total | 11(44) |

**Table 8** Mean postoperative hospital stay and return to daily activity

| *Group* | *Postoperative stay (days)* | *Return to activity (days)* |
|---|---|---|
| Uncomplicated (n = 201) | 12 | 61 |
| Complicated (n = 25) | 23 | 94 |
| | $p<0.001$ | $p<0.001$ |

**Table 9** Duration of drainage and hospital stay related to surgical procedures

| *Surgical procedure* | *Hospital stay (days)* | *Duration of drainage (days)* |
|---|---|---|
| Omentoplasty | 11.5 | 7.8 |
| External drainage of residual cavity | 12.5 | 9.2 |
| Marsupialization | 29.5 | 47 |
| Capitonnage | 13.6 | 11 |
| Introflexion | 10.5 | 7 |
| Pericystectomy | 14 | 13 |
| Hepatic resection | 15 | 13 |

### DISCUSSION

In endemic areas the diagnosis of hepatic hydatid cyst is no longer a difficult problem. After introducing organ imaging techniques; ultrasonography and computerised tomography, exact confirmation of cysts has become a much easier task[1,3]. In this study the imaging procedures ultrasonography and radionuclide scanning yielded very high percentage accuracy, 94% and 100% respectively.

The treatment for hydatid cysts is surgery. The principles of surgical management for hepatic echinococcosis, include (1) neutralisation of the parasite, (2) evacuation of the cyst and removal of the germinal lining, and (3) management of the residual cavity. Complete surgical removal which is the ideal treatment of the disease, can be accomplished by removal of all germinal lining, daughter cysts, fluid and scolices leaving the pericyst; or by resection of the intact cyst including pericyst[4]. At first, which method is used, cyst fluid should be neutralised to prevent accidental spillage of scolices or germinal lining into operative field. Numerous solutions such as, hypertonic saline solution, 2% formalin, 0.5% silver nitrate, 10% aqueous povidone iodine and formalin have been used as scolicidal agent. There is debate not only as to most effective scolicide but also about the necessity of scolicidal injection before cyst evacuation[5,6]. Instead of using scolicidal agent, a cryogenic cone has been developed to obtain entry to and evacuation of the cyst without fear of spillage into the peritoneal cavity[6]. We have no experience of this method or of the suction cone devised by Aarons and Kune[7]. The use of scolicidal agents has a long tradition but there is little evidence to justify their use and they are probably of negligible value in multivesicular cysts[5]. Perhaps the most important aspect of the manoeuvre is partial decompression of the cyst contents which are under tension. Recently 0.5% silver nitrate has become the solution of choice for many surgeons, including us. The solution of formalin causes sclerosan cholangitis have never been used in this series.

Because of the fear of spillage of cyst elements during surgery, medical treatment had been trained for hepatic hydatid cyst preoperatively and/or postoperatively. Lastly some reports have advocated the successful management of hydatid disease using benzimidazole compounds (mebendazole, albendazole) in small numbers of patients[8,9,10,11,12,13]. Because the general results are not reliable, medical treatment is not curative at present. Thus, these drugs should be reserved for patients who will not tolerate operation and, perhaps, for those with the more virulent form of alveolar hydatid disease of the liver caused by *Echinococcus multilocularis*.

The most difficult problem to be solved is the residual cavity in the treatment of hydatid cyst of the liver. After evacuation of cyst fluid all the simple communications between biliary tree and cyst cavity are open. This is the cause of biliary leakage postoperatively. A wide variety of techniques have been proposed to deal with the residual cavity after evacuation to prevent biliary leakage, biliary fistula and abscess. For these purposes, practised alternatives include: hepatic resection[2,14], percystectomy[15,16], omentoplasty[1,17,18], capitonnage[19], cystojejunostomy[20], marsupialisation, external drainage[21], introflexion[22], saucarisation and finally primary closure after saline instillation[23]. In selecting a particular surgical technique, the surgeon should be guided by the size and location of the cyst and the existence of complications.

Whenever possible, we managed the residual cyst cavity by omentoplasty. It has been shown that this technique reduces hospital stay and lowers the incidence of biliary fistula compared with marsupialization or tube drainage[17]. Our findings in 87 patients treated with omentoplasty confirm these satisfactory results. The omentum may obliterate the residual cavity and prevent secondary infection. (There seems advantage of omentoplasty to obliterate the cavities, as infection is quite uncommon.)

However, in some patients, the omentum may not be available because of previous operation or the technique cannot be performed because of very high and far location of the cyst. Furthermore, omentoplasty itself can cause formation of adhesions which, consequently, make difficult future operations upon the liver.

Also, introflexion is an alternative safe method for the treatment of the remaining cavity. It may be easily applied in most of the patients with cyst located periferally. This technique not only prevents dead space and its possible potential complications, but also covers the inner surface of the cystic cavity by several layers of peritoneum, which has been demonstrated to have a high resorptive capacity[22,24]. The omentoplasty resembles introflexion in that it enables the omentum to fill the cavity with its absorptive capacity; however, introflexion is most always applicable whereas omentoplasty is either difficult or impossible, at least in some instances[10]. These two methods can be used alternatively.

In the capitonnage method, the cystic walls are approximated by sutures to obliterate the cavity. Adjacent intrahepatic vessels may be injured; moreover, approximation may be very difficult or impossible in large cavities.

Marsupialization of the residual cavity has not been performed in our clinic for ten years, because its results have been unsatisfactory.

Because of the risk of spillage of infective material into the peritoneal cavity leading to recurrent disease, some authors have favoured resection[2,14], but this approach carries a significant operative risk and is not applicable in many cases[4]. Total cystectomy may be preferred for cysts located peripherally. Hepatic lobectomy and pericystectomy are, in our opinion, too radical and extensive procedures for a benign lesion.

Intrabiliary rupture is the commonest complication of hepatic hydatid cysts[5,25]. The pressure inside the cysts is always higher than the pressure in the biliary tract and after rupture the cyst elements pass into the biliary ducts. The liver cyst material does not die in the biliary channels and may cause obstruction and cholangitis at any stage of their life cycle[3,26]. Eight patients in this series (3.5 per cent) had cholangitis due to intrabiliary rupture of hydatid cyst.

The principles of operative management in these cases were to treat the mother cyst and to clear the biliary tree of any hydatid material. In this case intraoperative diagnosis is very important and there are two strategies of the operation. The first step is the surgical treatment of the cyst. The second is the exploration and drainage of the common bile duct. From the point of view of the intrabiliary rupture any technique can be used for the cyst cavity but the removal of all the cystic elements is the important point[3,26]. Drainage of the dilated common bile duct may be essential to avoid death from suppurative cholangitis or septic shock[3,26–29]. A drainage procedure, T tube[29–31], sphincterotomy[32], choledochoduodenostomy[3,26] or cystojejunostomy[21,31] should be added if there is any doubt about free biliary drainage.

In many large series of echinococcal liver cysts, operative mortality has ranged from 2 to 4 per cent, but has recently been reported to be as high as 6.3 per cent[14,17]. In our series, the mortality rate was 1.8 per cent.

From the result of this study, it is understood that close cavity with narrow stoma should not be left after evacuation of hydatid cyst in the liver. The cavity should be obliterated if it is possible. Omentoplasty and introflexion could be used alternatively to achieve obliterated cavity. Nevertheless, it is clear that complicated cysts cause more morbidity significantly than uncomplicated cysts. Therefore early diagnosis and early surgical treatment are essential for good results.

### *References*

1. Kune, G.A. (1985) Hydatid disease. In ĩMaingot's Abdominal Operations, edited by Schwartz, S.I., Ellis, H. 8th ed. pp. 1605–1624. Connecticut: Appleton - Century - Crofts
2. Pissiotis, C.A., Wander, J.U. and Condon, R.E. (1972) Surgical treatment of hydatid disease. Prevention of complications and recurrence. *Arch.Surg.*, **104**, 454–459
3. Bilge, A., Bengisu, N., Toyganözü, Y., Alper, A. (1988) Evaluation and treatment of patients with obstructive jaundice. *Japanese Journal of Medical Imaging*, **7**(1), 58–63
4. Langer, B. (1987) Surgical treatment of hydatid disease of the liver. *Br.J.Surg.* **74**, 237–238
5. Dawson, J.L., Stamatakis, J.D., Stringer, M.D. and Williams, R. (1988) Surgical treatment of hepatic hydatid disease. *Br.J.Surg.*, **75**, 946–950
6. Saidi, F. (1977) A new approach to the surgical treatment of hydatid cysts. *Ann.R.Coll,Surg.Engl.*, **59**, 115–128
7. Aarons, B.J. and Kune, G.A. (1983) A suction cone to prevent spillage during hydatid surgery. *Aus.NZ.Surg.*, **53**, 471–474
8. Muller, E., Ekovbiantz, A. and Ammann, R.W. (1982) Treatment of human echinococcosis with mebe dazole: preliminary observations in 28 patients. *Hepatogastroenterology*, **29**, 236–239
9. Musio, F. and Linos, D. (1989) Echonococcal disease in an Extended Family and Review of the Literature. *Arch.Surg.*, **124**, 741–744
10. Pitt, H.A., Korzelius, J. and Tompkins, R. (1986) Management of Hepatic Echonicoccosis in Southern California. *Am.J.Surg.* **152**, 110–115
11. Ronconi, P., Boraone, A. and Alquati, P. (1982) Preoperative treatment of hydatid cysts with mebendazole. *Int.Surg.*, **67**, 405–406
12. Saimod, A.G., Meulemans, A. and Cremieux, A.C. (1983) Albendazole as a potential treatment for human hydatidosis. *Lancet*, **2**, 652–656
13. Smego, D.R. and Smego, R.A.Jr, (1986) Hydatid cyst, preoperative sterilization with mebendazole. *South.Med.J.*, **79**, 900–901
14. Belli, L., Favero, E., Marni, A. and Romani, R. (1983) Resection versus pericystectomy in the treatment of hydatidosis of the liver. *Am.J.Surg.*, **145**, 239–242
15. Placer-Galan, C., Martin, R., Jimenez, R. and Soleto, E. (1987) A simplified technique for surgical management of echinococcal cyst. *Surg.Gynecol.Obstet.*, **165**, 269–270
16. Belli, L., Romani, F. and Puttini, M. (1987) Easier and safer cystopericystectomy using the pihgle maneouvre. *Surg.Gynecol.Obstet.*, **164**, 75–76
17. Papadimitriou, J. and Mandrekas, A. (1970) The surgical treatment of hydatid disease of the liver. *Br.J.Surg.*, **57**, 431–433
18. Little, J.M. and Deane, S.A. (1986) Hydatid disease. In *Liver Surgery*, edited by Bengmark, S., Blumgart, L.H. pp. 118–129. Edinburgh: Churchill Livingstone

19. Akinoğlu, A., Bilgin, I. and Erkoçak, E.U. (1985) Surgical management of hydatid disease of the liver. *Can.J.Surg.*, **28**, 171–174
20. Sekar, N., Mahajan, K.K. Kaushik, S.P. and Katariya, R.N. (1982) Pericystojejunostomy in the treatment of hydatid cysts of the liver. *Aus.NZ.J.Surg.*, **52**, 76–78
21. Barros, J.L. (1978) Hydatid disease of the liver. *Am.J.Surg.*, **135**, 597–600
22. Arioğul. O., Emre, A., Alper, A. and Uras, A. (1989) Introflexion as a method of surgical treatment for hydatid disease. *Surg.Gynecol.Obstet.*, **169**, 356–358
23. Ekrami. Y. (1976) Surgical treatment of hydatid disease of the liver. *Arch.Surg.*, **111**, 1350–1352
24. Condon, R.E. and Malangoni, M.A. (1984) Peritonitis and intraabdominal abscesses. In *Principles of Surgery*. Edited by Schwartz, S.I., Shires, G.T., Spencer, F.C., Storer, E.H., pp. 1391–1392. New York: McGraw-Hill Book Co
25. Androulakis, G.A. (1986) Surgical management of complicated hydatid cysts of the liver. *Eur.Surg.Res.*, **18**, 145–150
26. Alper, A., Arioğul, O., Emre, A., Uras, A. and Ökten, A. (1987) Choledochoduodenostomy for intrabiliary rupture of hydatid cysts of liver. *Br.J.Surg.*, **74**, 243–245
27. Cottone M., Amuso, M. and Cotton, P.B. (1978) Endoscopic retrograde cholangiography in hepatic hydatid disease. *Br.J.Surg.*, **65**, 107–108
28. Ovnat, A., Peiser, J., Avinoah, E., Barki, Y. and Charuzi, I. (1984) Acute cholangitis caused ruptured hydatid cyst. *Surgery*, **95**, 497–500
29. Sayek, I., Yalin, R. and Sanaç, Y. (1980) Surgical treatment of hydatid disease of the liver. *Arch.Surg.*, **115**, 847–850
30. Dadoukis, J., Gamvros, O. and Aletras, H. (1984) Intrabiliary rupture of the hydatid cyst of the liver. *World.J.Surg.*, **8**, 786–790
31. Lygidakis, N.J. (1983) Diagnosis and treatment of intrabiliary rupture of hydatid cyst of the liver. *Arch.Surg.*, **118**, 1186–1189
32. Moveno, V.F. and Lopez, E.V. (1985) Acute cholangitis caused by ruptured hydatid cyst (letter). *Surgery*, **97**, 249–250

*(Accepted by S. Bengmark 13 September 1990)*

